# Metabolic reprogramming of the myeloid lineage by *Schistosoma mansoni* infection persists independently of antigen exposure

**DOI:** 10.1371/journal.ppat.1009198

**Published:** 2021-01-08

**Authors:** Diana Cortes-Selva, Lisa Gibbs, J. Alan Maschek, Marcia Nascimento, Tyler Van Ry, James E. Cox, Eyal Amiel, Keke C. Fairfax

**Affiliations:** 1 Department of Pathology, Division of Microbiology and Immunology, University of Utah, Salt Lake City Utah, United States of America; 2 Department of Comparative Pathobiology, College of Veterinary Medicine, Purdue University, West Lafayette Indiana, United States of America; 3 Metabolomics, Proteomics and Mass Spectrometry Cores, University of Utah, Salt Lake City, Utah, United States of America; 4 Department of Nutrition and Integrative Physiology and the Diabetes and Metabolism Research Center, University of Utah, Salt Lake City, Utah, United States of America; 5 Department of Biochemistry, University of Utah, Salt Lake City Utah, United States of America; 6 Department of Biomedical and Health Sciences, University of Vermont, Burlington, Vermont, United States of America; University of Pennsylvania School of Veterinary Medicine, UNITED STATES

## Abstract

Macrophages have a defined role in the pathogenesis of metabolic disease and cholesterol metabolism where alternative activation of macrophages is thought to be beneficial to both glucose and cholesterol metabolism during high fat diet induced disease. It is well established that helminth infection protects from metabolic disease, but the mechanisms underlying protection are not well understood. Here, we investigated the effects of *Schistosoma mansoni* infection and cytokine activation in the metabolic signatures of bone marrow derived macrophages using an approach that integrated transcriptomics, metabolomics, and lipidomics in a metabolic disease prone mouse model. We demonstrate that bone marrow derived macrophages (BMDM) from *S*. *mansoni* infected male ApoE^-/-^ mice have dramatically increased mitochondrial respiration compared to those from uninfected mice. This change is associated with increased glucose and palmitate shuttling into TCA cycle intermediates, increased accumulation of free fatty acids, and decreased accumulation of cellular cholesterol esters, tri and diglycerides, and is dependent on mgll activity. Systemic injection of IL-4 complexes is unable to recapitulate either reductions in systemic glucose AUC or the re-programing of BMDM mitochondrial respiration seen in infected males. Importantly, the metabolic reprogramming of male myeloid cells is transferrable via bone marrow transplantation to an uninfected host, indicating maintenance of reprogramming in the absence of sustained antigen exposure. Finally, schistosome induced metabolic and bone marrow modulation is sex-dependent, with infection protecting male, but not female mice from glucose intolerance and obesity. Our findings identify a transferable, long-lasting sex-dependent reprograming of the metabolic signature of macrophages by helminth infection, providing key mechanistic insight into the factors regulating the beneficial roles of helminth infection in metabolic disease.

## Author summary

Globally, helminth endemic regions have lower incidences of metabolic disease. Schistosomiasis in particular has been shown to protect male mice from the development of atherosclerosis, diabetes and obesity while altering the metabolism of liver macrophages. In this study we sought to understand if metabolic modulation occurs systemically. We have found for the first time that macrophages generated from bone marrow myeloid progenitors from infected male mice have long-lived increases in their basal metabolism of lipids. We have also found the type-2 polarizing cytokine IL-4 is not sufficient to replicate infection induced metabolic reprogramming. Importantly, we demonstrate that the changes to male macrophage metabolism can be transferred to an uninfected host. This suggests that metabolic reprogramming is long-lived without exposure to active infection, and develops a memory-like phenotype in myeloid cells.

## Introduction

Cardiovascular disease (CVD) is the leading worldwide cause of mortality [1, 2]. In the United States, 65% of adults diagnosed with diabetes have elevated LDL cholesterol levels or take cholesterol lowering medications, and death rates from atherosclerotic cardiovascular disease (CVD) are ~1.7 times higher in this population as compared to non-diabetic adults [3]. It is well established that in the diabetic population, obesity, and dyslipidemia are risk factors underlying these increases in mortality, while hyperglycemia is an independent risk factor [4, 5]. Insulin resistance is associated with increased free fatty acids in the plasma, leading to increased insulin and hepatic glucose production in type-2 diabetic patients [6, 7]. Recent studies report that intensive treatment of hyperglycemia, when initiated early during the course of diabetes, can result in cardiovascular benefits, a process postulated to result from “metabolic memory” and epigenetic changes [8–10].

Previous studies have uncovered an association between a history of helminth infection and reduced prevalence of metabolic disease in humans and rodents [11–13]. Specifically, infection by schistosomes reduces serum cholesterol and atherosclerotic plaques [11, 12]. This effect has been attributed, in part, to an anti-inflammatory phenotype in macrophages [14] and transcriptional reprogramming of phospholipid and glucose metabolism related genes in hepatic macrophages [15]. *Schistosoma mansoni* egg antigen exposure has previously been shown to be required for schistosomiasis induced modulation of systemic metabolism [11].

Schistosomiasis is a systemic infection that is known to induce an early IFNγ response that transitions to a mixed response where IL-4 and IL-13 production is greater than IFNγ by 10-weeks post infection [16, 17], and IL-4Rα driven alternative activation of macrophages is essential for host survival, as mice that lack signaling die of acute disease [18–20]. *S*. *mansoni* has also been shown to induce monopoiesis and monocytosis to meet the increased need for hepatic macrophages [21]. IL-4 induced alternative activation of macrophages relies on oxidative phosphorylation (OXPHOS) and fatty acid oxidation for energy production, and has been shown to be dependent on cell intrinsic lysosomal lipolysis [22, 23].

In the present study, we sought to determine the systemic effects of *S*. *mansoni* infection on the myeloid lineage in the context of high fat diet (HFD) induced metabolic disease. Surprisingly, we discovered that macrophages derived from the bone marrow of *S*. *mansoni* infected male Apolipoprotein E deficient (ApoE^-/-^) mice on HFD have dramatically increased oxygen consumption and mitochondrial mass compared to those from uninfected males. This shift is accompanied by a decrease in cellular cholesterol esters, and di/triglycerides; and increases in cellular free fatty acids and fatty acid oxidation. Injection of IL-4 complexes to simulate the induction of IL-4 by egg antigens, failed to recapitulate the infection induced reprogramming of macrophage respiration. Infection induced metabolic reprograming of myeloid cells is long-lived in the absence of antigen, and protection from glucose intolerance is transferrable to an uninfected host via bone marrow transfer. Surprisingly, systemic protection from the metabolic disease parameters of obesity and glucose intolerance is sex-dependent, as *S*. *mansoni* infection fails to protect female ApoE^-/-^ mice from disease. Overall, these data present the first evidence that *S*. *mansoni* systemically modulates the myeloid compartment and provide a more comprehensive understanding of how *S*. *mansoni* infection may confer metabolic protection distinctly from IL-4, at the cellular level.

## Results

### Macrophages derived from *S*. *mansoni* infected male mice have increased oxygen consumption and spare respiratory capacity

**Fig 1:**
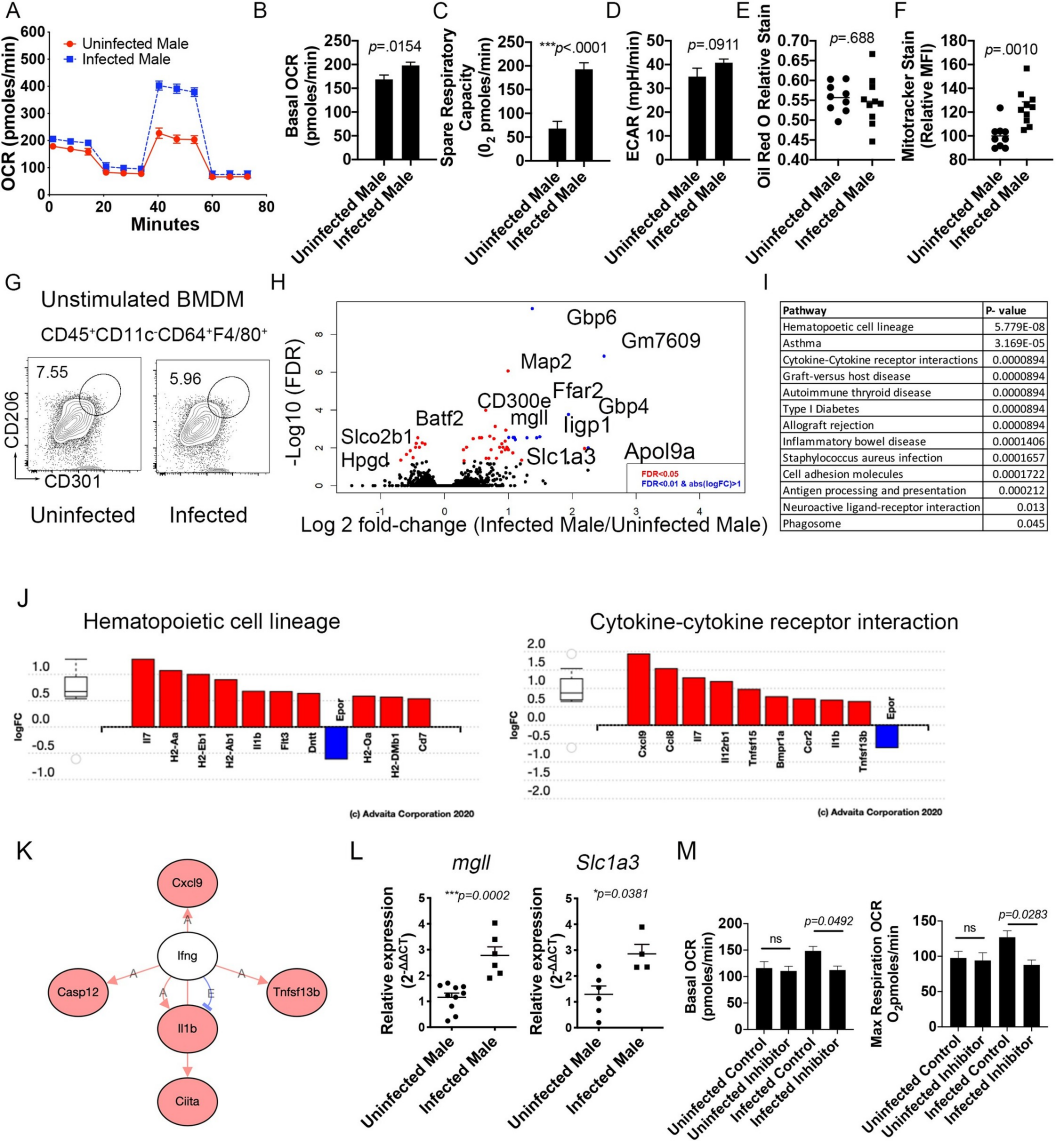
Bone marrow derived macrophages (BMDM) from ApoE^-/-^ male *S*. *mansoni* infected mice exhibit increased oxygen consumption and mitochondria mass. ApoE^-/-^ male were fed HFD for 10 days before infection with *S*. *mansoni*. Ten weeks post infection mice were sacrificed and bone marrow cells were harvested and cultured under M-CSF. (A) SeaHorse assay results for OCR of BMDM from infected and uninfected ApoE^-/-^ males in basal conditions and in response to mitochondrial inhibitors. (B) Quantification (in picomoles/minute) of the basal oxygen consumption of BMDM. (C) Quantification of the spare respiratory capacity of BMDM (D) Extracellular acidification rate of BMDM. (E) Oil Red O relative staining in BMDM. (F) MitoTracker Red Deep Stain measured by flow cytometry in BMDM. (G) Flow cytometry of alternative activated markers in unstimulated BMDM. (H) Volcano plot of significantly differentially expressed genes between BMDM from *S*. *mansoni* infected uninfected mice. I) iPathway analysis showed distinct profiles in BMDM from *S*. *mansoni* infected mice. J) Differentially regulated genes in the Hematopoetic cell lineage and cytokine-cytokine receptor interactions pathways as analyzed in iPathway analysis. K) Interactome diagram of the genes differentially regulated by S. mansoni infection downstream of IFNγ. L) Real-time PCR validation of *mgll* and *slc1a3* regulation in BMDM. M) Quantitation of basal and maximum Respiration OCR of BMDM from infected and uninfected control ApoE-/- mice with and without the mgll inhibitor JZL 184. SeaHorse assay analysis were performed the Seahorse XFe96 instrument. *p < 0.05; **p < 0.01; ***p < 0.001. Graphs are representative of multiple experiments (2–3), with n>4 per group. Data in J and K represent 2 biologically independent experiments with 4–6 mice per group, data in M are from 1 experiment with 4–5 mice per group.

We have previously reported that schistosomiasis alters the expression of numerous genes relevant to glucose, cholesterol, and amino acid metabolism in hepatic macrophages of male mice [15]. These alterations are associated with improved insulin sensitivity and atherosclerotic score in mice. Since it has previously been shown that during *S*. *mansoni* infection the majority of liver macrophages are monocyte derived, and monocyte recruitment drives both atherosclerosis [24, 25] and obesity induced insulin resistance [26–29], we hypothesized that schistosome infection may imprint the monocyte- macrophage progenitor cell lineage with altered metabolic potential. To elucidate whether infection imprints macrophages with an altered metabolic phenotype we infected (and mock infected controls) atherogenesis-prone male ApoE^-/-^mice on HFD, and sacrificed them at 10-weeks post-infection (chronic infection) to harvest bone marrow cells. Macrophages were differentiated *in vitro* with M-CSF in a 6-7-day culture. We performed real-time extracellular flux analysis on unstimulated bone marrow-derived macrophages (BMDM) from ApoE^-/-^ HFD infected and uninfected (control) mice to quantify oxygen consumption rate (OCR) (Fig 1A). BMDM from ApoE^-/-^ HFD infected mice showed higher basal respiration (Fig 1B) and significantly increased spare respiratory capacity (p<0.0001, Fig 1C). Since eukaryotic cells integrate oxidative phosphorylation (OXPHOS), glycolysis and the tricarboxylic acid (TCA) cycle to satisfy energy requirements, we also tested the extracellular acidification rate (ECAR), which has previously been linked to the development of inhibited mitochondrial respiration [30, 31], in BMDM from infected and uninfected ApoE^-/-^ HFD mice. We observed no differences in ECAR in infected male mice compared to uninfected controls (Fig 1D). Cell intrinsic lysosomal lipolysis has previously been shown to support macrophage spare respiratory capacity in the context of alternative activation [23, 32]. We stained for hydrophobic and neutral lipids by Oil Red O (ORO) [33] and observed that the lipid content of BMDM from infected ApoE^-/-^ males was not significantly reduced (Fig 1E). To analyze BMDM mitochondrial mass, which has also been linked to increased respiratory capacity [34], we analyzed mitochondrial activity by Mitotracker Deep Red FM. We observed that BMDM from infected mice exhibited increased MitoTracker median fluorescent intensity (MFI) in comparison to the BMDM from uninfected mice (Fig 1F). Importantly, we found no upregulation of alternative activation markers (CD301, CD206) in unstimulated BMDM from infected mice when compared to BMDM from controls (Fig 1G). Next, we determined the transcriptomic modifications induced by infection in unstimulated BMDM from infected and uninfected males by mRNA sequencing (mRNASeq). Significant gene expression differences were observed in BMDM from infected male mice, compared to uninfected controls. Transcripts from the two groups are depicted in Volcano plots, using false discovery rate (FDR<0.05 in red, FDR<0.01 in blue) and Log2 fold changes (cut off of .6 Log2 FC) to identify statistically significant genes (Fig 1H). Among the differentially regulated factors was Mgll, which encodes monoacylglycerol lipase that catalyzes the conversion of monoacylglycerides to free fatty acids and glycerol, and was significantly increased. Previous work in a HFD model of obesity has shown that Mgll is required for lipolysis and improved glucose homeostasis in mice on HFD [35]. In addition, Slc1a3, which encodes for the glutamate aspartate transporter 1 that is localized in the inner mitochondria membrane as part of the malate-aspartate shuttle and is relevant for amino acid homeostasis in adipocytes, was significantly altered in our model. Interestingly, the pathways significantly altered (Fig 1I) during infection included hematopoietic cell lineage (p = 5.779 x10^-8^), asthma (p = 3.169 x10^-5^), and cytokine-cytokine receptor interactions, graft-versus host disease, type 1 diabetes and allograft regression (p = 8.94 x10^-5^). Analyzing the differentially regulated genes within the hematopoietic and cytokine- cytokine receptor pathways (Fig 1J), we found significant upregulation of *il1b*, *flt3*, *ccr2*, and *il12rb1*, suggesting that BMDM from infected males have some transcriptomic aspects of a proinflammatory gene signature without an increase in extracellular acidification. Intriguingly, multiple genes downstream of IFNγ signaling are upregulated in BMDM from infected males, without an increase in IFNγ itself (Fig 1K). Upregulation of both Slc1a3 and Mgll expression was further validated by RT-qPCR (Fig 1L). Since upregulation of mgll is one way that BMDM from infected males may generate substrates for mitochondrial β-oxidation, we employed JZL 184, a commercially available inhibitor of mgll in a SeaHorse assay. Pre-incubation with 1μM JZL 184 significantly reduced basal and maximal OCR in BMDM from infected male mice, but not from uninfected males. These data support the premise that infection induced increases in fatty acid oxidation seen in Fig 1A are driven at least in part by fatty acids released via mgll activity. Overall, these data indicate that *S*. *mansoni* infection in males leads to increased oxygen consumption and mitochondrial metabolism in BMDM, while inducing upregulation of acute proinflammatory transcripts that have previously been associated with aerobic glycolysis induced mitochondrial dysfunction. Mitochondrial oxidative dysfunction in macrophages has recently been linked to insulin resistance [36], so this metabolic shift in the myeloid hematopoietic compartment could contribute to the infection-induced improvement in glucose tolerance seen in males, via preservation of mitochondrial function.

### Schistosomiasis in male ApoE^-/-^ mice alters metabolic flux of glucose and the lipidomic fingerprint of macrophages

**Fig 2:**
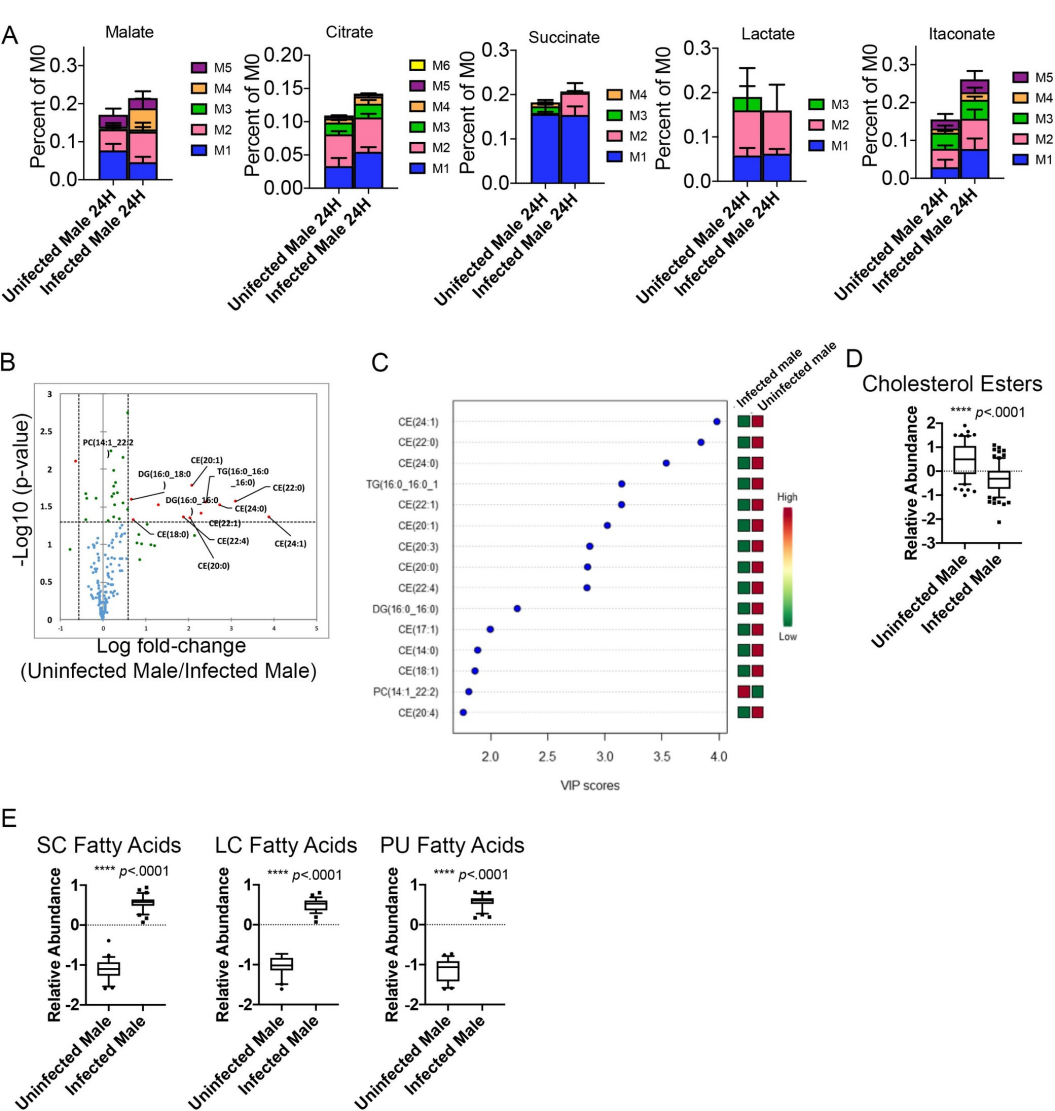
BMDM from male *S*. *mansoni* infected HFD ApoE^-/-^ mice have increased TCA cycle usage with significantly increases free fatty acids and reduced cholesterol esters. A-E) Mϕ were differentiated from bone marrow of 10-week infected animals with M-CSF in a 7-day culture in normal glucose and then switched to ^13^C-labeled glucose for 24 hours. F-I) Mϕs were differentiated with M-CSF in a 7-day culture and then total cellular lipids were extracted and analyzed via LC-MS based lipidomic analysis. B) Volcano plot of significantly altered species in uninfected: infected male BMDM C) The variable importance in projections (VIP) scores from uninfected males in comparison to infected males D-E) Box whiskers plots (10–90 percentile) of relative abundance of normalized cholesterol ester and the indicated free fatty acid species, data are normalized to sum and treated with pareto scaling, each dot is a single species. Data are representative of 3 experiments with 4–6 mice per group in each experiment.

In order to understand how schistosome infection alters the metabolic fingerprint and promotes mitochondrial metabolism in macrophages derived from ApoE^-/-^ HFD male mice, we performed metabolic tracing analysis, where unstimulated macrophages were differentiated in the presence of normal glucose and then switched to ^13^C-labeled glucose for 24 hours. We observed increased shuttling of heavy labeled glucose to malate carbon position 4 (*p* = .0476), succinate (carbon position 2 *p* = .0159), and itaconate carbon positions 1 and 2 (*p* = .0405), while shuttling into citrate trended higher, but was not significant (Fig 2A) in BMDM from infected mice in comparison to BMDM from uninfected mice. The lack of increased heavy lactate production (Fig 2A), suggests that the primary reprogramming is focused on glucose-dependent mitochondrial metabolism. Since we have previously shown that infection alters phospholipid and cholesterol metabolism in hepatic macrophages [15], we performed untargeted lipidomics using LC-MS. The lipid species significantly altered by infection (*p*-value < 0.05 and a fold change (FC) ≥1.5) are depicted on a volcano plot (Fig 2B). Twelve of the fifteen lipids that meet this threshold are cholesterol esters (Fig 2C). We then analyzed the relative abundance of cholesterol esters (CE) as a class in macrophages from both groups of mice, and further confirmed that infection led to significantly reduced CEs in male mice (p<0.0001, Fig 2D), further evidencing an important role of cholesterol metabolism in macrophages following helminth infection. Since the extracellular flux data suggested increased mitochondrial fatty acid oxidation (FAO), and the glucose tracing data indicated flux of heavy labeled carbons through the TCA cycle (a possible pathway for generating palmitate *de novo* from acetyl-CoA), we examined the relative abundance of free fatty acids (possible substrates for FAO) in BMDM. Infection drives significant increases in cellular long chain (LC), short chain (SC), and polyunsaturated (PU, both SC and LC) free fatty acids (Fig 2E).

### *S*. *mansoni*-increases fatty acid oxidation in male ApoE^-/-^ mice on HFD

**Fig 3:**
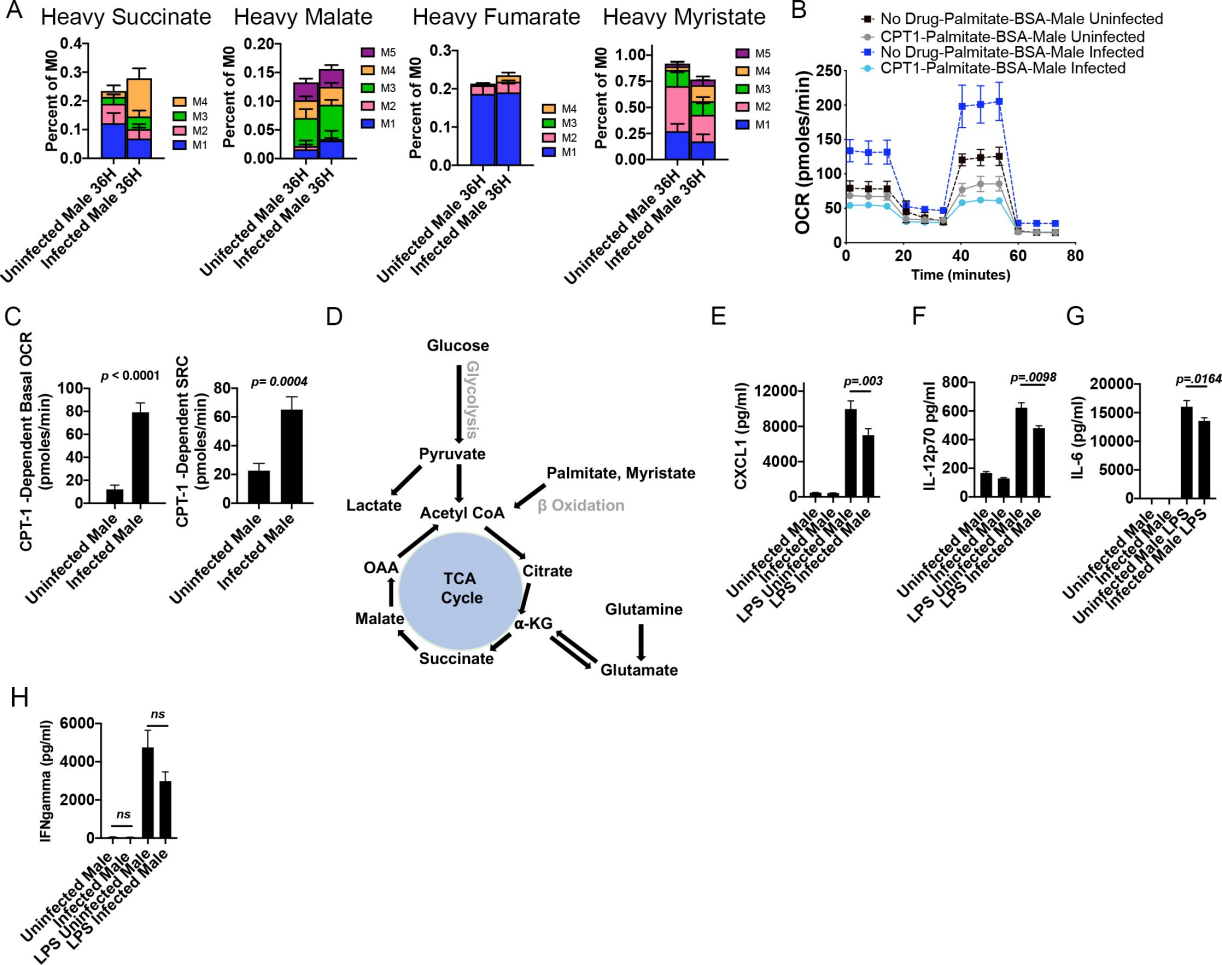
*S*. *mansoni* infection induces fatty acid oxidation and lowers chronic inflammatory factors in male ApoE^-/-^ male mice. (A) Macrophages from control and *S*. *mansoni* infected ApoE^-/-^ mice were differentiated with M-CSF in a 7-day culture with normal glucose and serum, and then switched to ^13^C-labeled palmitate in dialyzed serum for 36 hours followed by metabolic flux analysis (B-C) BMDM were differentiated with M-CSF in a 7-day culture with normal glucose and serum, and then switched to glucose limited media with Palmitate-BSA as the carbon source followed by Seahorse analysis of OCR and SRC in the presence of the CPT-1 inhibitor etomoxir. (D) Schematic of glucose, glutamine, and palmitate inputs into the TCA cycle. (E-H) Quantification of inflammatory mediators in BMDM supernatants 24 hours post stimulation with LPS. Data are representative of two biologically independent experiments.

Since we had observed both an increase in spare respiratory capacity and cellular free fatty acids in BMDM from infected males, we directly assessed the uptake and usage of exogenous fatty acids through metabolic tracing analysis of palmitate. Macrophages were differentiated from infected and uninfected BM in the presence of normal glucose and serum, and then switched to ^13^C-labeled palmitate conjugated to BSA in dialyzed serum for 36 hours. Similar to what we documented with glucose, we observed increased shuttling of heavy labeled palmitate into succinate, malate, and fumarate in BMDM derived from infected males (Fig 3A). BMDM from infected males had a significant increase in incorporation into succinate at carbon positions 3 and 4 (*p* = 0.0123) and fumarate at carbon positions 2,3, and 4 (*p* = 0.0447) and a trend towards increased incorporation in malate. This was accompanied by decreased shuttling into myristate (*p* = 0.0221). These data suggest that carbons from the fatty acid palmitate have a similar shuttling through the TCA cycle as those from glucose. In order to quantify the effect of this uptake of exogenous palmitate on fatty acid oxidation we measured OCR in real time in glucose limited conditions with palmitate conjugated to BSA as the carbon source, with and without the CPT-1 inhibitor etomoxir [23, 37]. BMDM from infected males had an increased ability to oxidize exogenous palmitate (Fig 3B) as compared to BMDM from uninfected males, and this increased usage of exogenous palmitate was dependent on CPT-1, as addition of etomoxir significantly decreased both basal oxygen consumption and SRC (Fig 3B). Specifically examining the CPT-1 dependent respiration, BMDM from infected males had significantly increased CPT-1 dependent Basal OCR and SRC (Fig 3C). In order to confirm that infection induced enhanced oxygen consumption and spare respiratory capacity was not confined to the ApoE^-/-^ genetic background, we assayed exogenous palmitate respiration in BMDM from IL4R^Fl/Fl^Cre^neg^ mice (S1 Fig). Similar to the data from ApoE^-/-^ mice, BMDM from infected IL4R ^Fl/Fl^Cre^neg^ males have significantly higher basal OCR and SRC that BMDM from uninfected animals in conditions where palmitate is the sole exogenous carbon source (S1B and S1C Fig). Additionally, BMDM from infected males have significantly higher mitotracker MFI with no significant difference in total neutral lipids as measured by oil red O staining (S1D and S1E Fig). Fig 3D depicts how glucose and fatty acids like palmitate can cycle through the TCA cycle to meet the ATP demands of a cell. Cholesterol and lipid metabolism has previously been associated with inflammatory myeloid effector function [38–40], so we quantified the pro-inflammatory cytokines/chemokines IL-12p70, CXCL1, and IL-6 (chemokines/cytokines with known pathogenic roles in obesity, insulin resistance and atherosclerosis) following stimulation with LPS. BMDM from infected males have decreased production of IL-2p70, CXCL1, and IL-6 but no change in IFNγ (Fig 3E–3H) following LPS stimulation as compared to BMDM from uninfected controls. This decrease in mediators of chronic inflammation supports the idea that infection induced reprograming of lipid metabolism may underlie macrophage effector function during metabolic disease.

### Systemic IL-4 administration does not phenocopy infection induced metabolic re-programing

**Fig 4:**
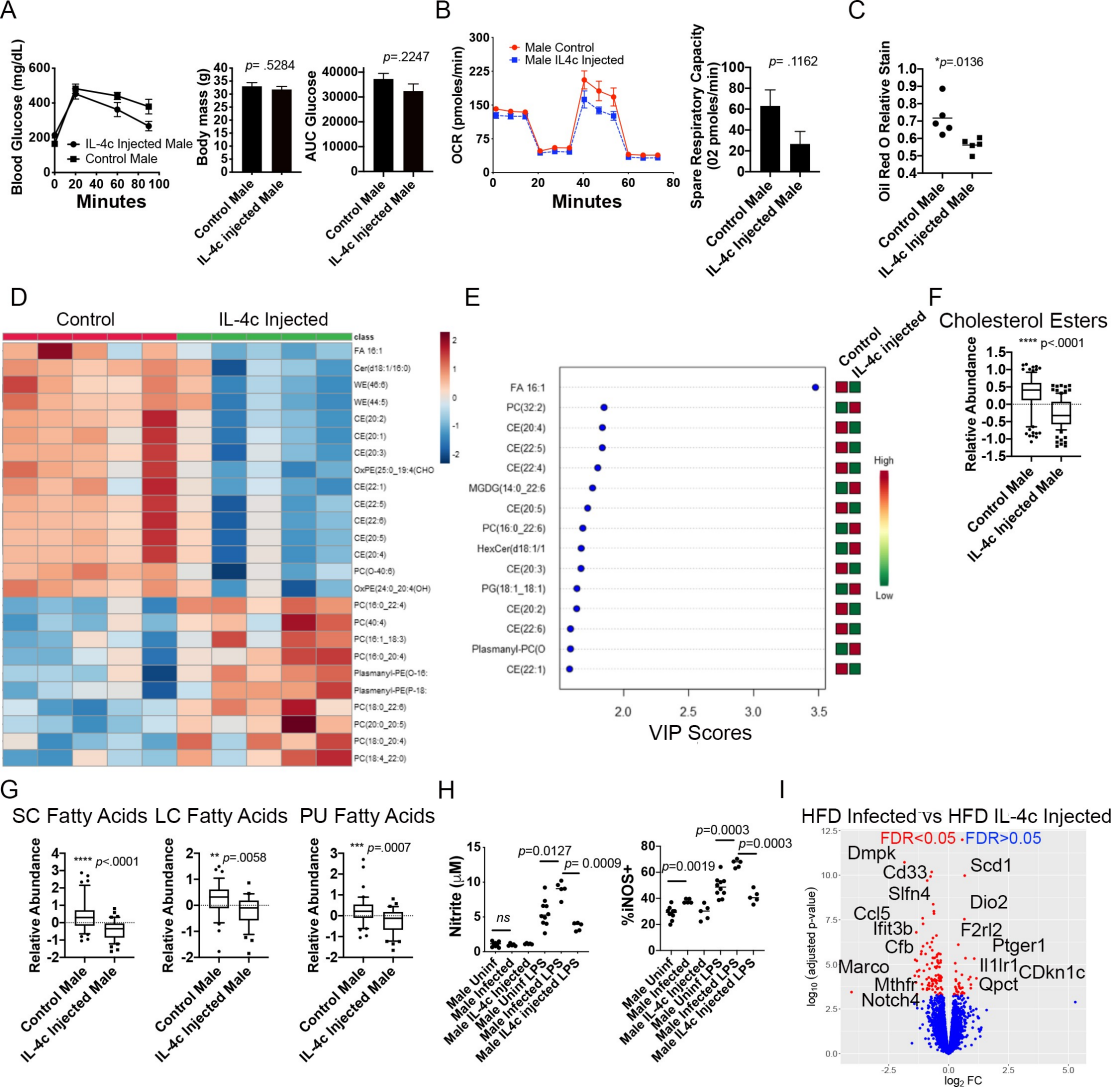
Systemic IL-4 complexes do not protect from HFD-induced weight gain or phenocopy *S*. *mansoni* induced BMDM re-programing. ApoE^-/-^ mice on HFD were injected with IL-4 complexes or PBS control for 4–6 weeks. (A) Glucose tolerance test and body weight measurements were performed in ApoE^-/-^ mice at 6–8 weeks post HFD. (B and C) Oxygen consumption rate and spare respiratory capacity were measured at steady state in BMDM from IL-4c injected and control mice. (D) Heat map of 25 top species differentially regulated by systemic IL-4 administration (E) VIP scores from the PLS-DA analysis between control PBS injected and IL-4c treated mice (F,G) Box whiskers plots (10–90 percentile) of relative abundance of cholesterol esters and the indicated free fatty acid classes (data are normalized to sum and treated with pareto scaling, each dot is a single species). (H) Culture supernatants from BMDM stimulated with LPS for 24 hours were assayed for nitrate and iNos (I) Volcano plot of significantly differentially expressed genes between BMDM from *S*. *mansoni* infected and IL-4c treated ApoE^-/-^ mice. Graphs are representative of 2 biologically independent experiments with 5–8 mice per group.

The metabolically protective effects of helminth infections have previously been suggested to be driven by systemic type-2 polarization. IL-4 and alternative activation of hepatic macrophages are essential to host survival during *S*. *mansoni* infection [19, 41, 42], so it is not possible to block IL-4 signaling in infected ApoE^-/-^ mice on HFD. Instead, we examined the sufficiency of chronic systemic IL-4 to induce reprogramming of BMDM by placing male ApoE^-/-^ mice on HFD for 4–5 weeks and then administering IL-4 complexes (IL-4c, [43]) to ApoE^-/-^ mice on HFD for ~4.5 weeks to mimic the time period of egg antigen exposure in our infection model (egg laying begins at 6 weeks post *S*. *mansoni* infection) and then harvested bone marrow. This period of IL-4c administration was sufficient to induce M2 polarization of macrophages in the peritoneal cavity (S2A Fig). IL-4c injection did not improve obesity or glucose tolerance in HFD ApoE^-/-^ males (Fig 4A). We then differentiated macrophages *in vitro* with M-CSF in a 6-7-day culture and performed real-time extracellular flux analysis on unstimulated BMDM from IL-4c injected and control ApoE^-/-^ mice. Unlike BMDM from infected males (Fig 1), BMDM from IL-4c injected mice had equivalent OCR and SRC to BMDM from control males (Fig 4B and 4C), indicating that chronic *in vivo* IL-4 exposure is not sufficient to reprogram the mitochondrial respiration of BMDM. We then performed unbiased lipidomics using LC-MS. Analyzing the top 25 significantly changed compounds (determined by a t test), we find that chronic IL-4c injection predominately leads to reduced wax monoesters and cholesterol esters, but no decrease in triacylglycerol (TG), or diacylglycerol (DG) (Fig 4D). We further analyzed the lipidomes of IL-4c and control BMDM using a PLS-DA with two components. There was a separation between groups that indicates the metabolic profiles of BMDM from IL-4c-injected and control males on HFD differ significantly, and suggests that there is a prominent alteration of metabolites induced by chronic IL-4 exposure in male mice (S2B Fig). The lipid species that drive the variation observed in the PLS-DA as measured by the VIP score from control males in comparison to IL-4c treated males included decreased cholesterol esters, increases in two species of phosphatidylcholines and a phosphatidylglycerol, and decreased palmitoleic acid (Fig 4E). We then analyzed abundance of cholesterol esters and free fatty acids at the class level. While IL-4 injection induced a decrease in cholesterol esters similar to *S*. *mansoni* infection (Fig 4F), IL-4c injection induced the significant reduction in free SC, LC, and PU fatty acids (Fig 4G), the opposite of the metabolic reprogramming seen in BMDM from infected males. Since *de novo* synthesis of cholesterol has recently been demonstrated to be involved in LPS induced TLR4 signaling [40], we measured the downstream effector molecules iNos and nitrite. Surprisingly, infection significantly increased both unstimulated and LPS induced production of iNos, but only LPS induced production of nitrite, while chronic IL-4 exposure had no effect on the production of either metabolite (Fig 4H). These analyses indicate that *S*. *mansoni* infection, and chronic IL-4 exposure induce distinctly different modulations to the lipidomes and inflammatory potential of BMDM and the inflammatory potential fits with the increases in *il-1b* transcript seen in Fig 1. In order to understand the transcriptional changes that correlate with these metabolic phenotypes, we cultured BMDM from 10-week *S*. *mansoni* infected, IL-4c injected, and control ApoE^-/-^ mice on HFD for 7 days in M-CSF and then extracted RNA for mRNASeq analysis. Direct comparison of the BMDM transcriptomes from *S*. *mansoni* infected and IL-4c injected animals with a stringent FDR adjusted *p* value shows 81 genes have a log_2_FC > .5 and an FDR <0.05 (Fig 4I), multiple of these genes are involved in inflammation, methylation, and metabolism, including Ptger1, Marco, Mthfr, Scd1 and Dio2. Mthfr is downregulated in BMDM from infected males and upregulated in IL-4 injected, while Scd1 is upregulated in infected males and downregulated by IL-4 injection. The canonical M2 gene Chl3 (Ym1) is upregulated in BMDM from IL-4c injected mice (1.049 log_2_FC, FDR 1.17x10^-6^), but unchanged in BMDM from *S*. *mansoni* infected mice. iPathway analysis (Advaita) of the transcriptome of IL-4 injected HFD ApoE^-/-^ mice suggested a distinct transcriptional landscape from what is induced by *S*. *mansoni* infection (S2C and S2D Fig) with infection modulating more genes in metabolic pathways of BMDM than IL-4c injection.

### *S*. *mansoni* induced modulation of male macrophage metabolism is long-lived in the absence of antigen

**Fig 5:**
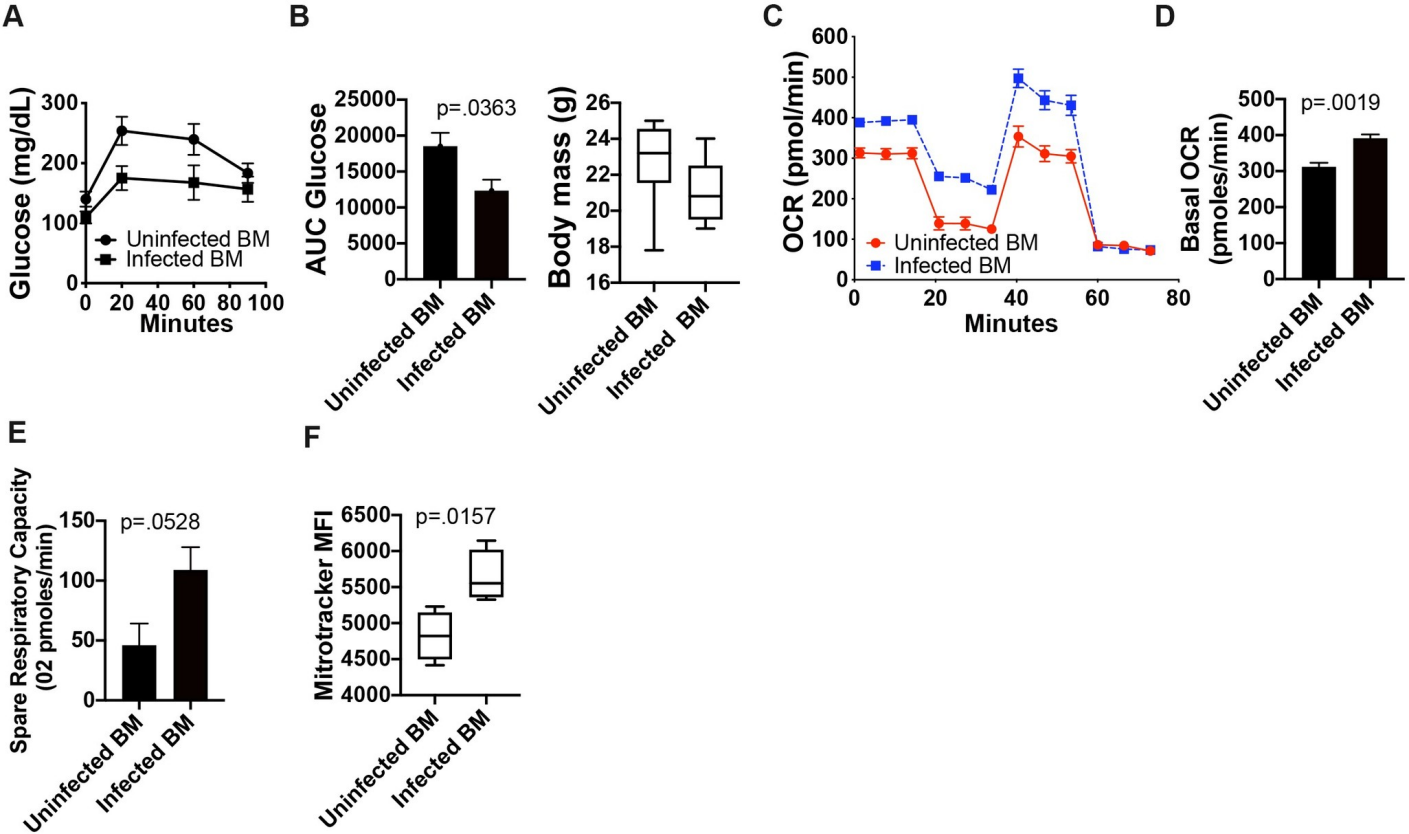
*S*. *mansoni* induced modulation of male macrophage metabolism is long-lived in the absence of antigen. Bone marrow from 10-week *S*. *mansoni* infected or control ApoE^-/-^ mice on HFD was transferred into busulfan treated ApoE^-/-^ recipients on HFD. A,B) Glucose tolerance test (GTT) and total body weight at 10 weeks post-transfer. GTT values were analyzed by Area Under the Curve using GraphPad Prism. C-E) OCR and spare respiratory capacity were measured at steady state in 7-day BMDM. F) MitoTracker Red Deep Stain measure by flow cytometry in BMDM. Data is two combined experiments with 7–8 animals per group. Statistical analysis was done using Welch’s t-tests.

Our metabolic and transcriptomic data from BMDM differentiated *in vitro* from male *S*. *mansoni* infected mice suggested that metabolic modulation may be long-lived in the absence of ongoing antigen exposure. In order to determine the durability of metabolic reprogramming, we transferred bone marrow from either 10-week *S*. *mansoni* infected male ApoE^-/-^ mice on HFD, or uninfected male controls into busulfan treated recipient ApoE^-/-^ mice on HFD. At 10 weeks post-bone marrow transfer we assayed glucose tolerance via an i.p. glucose tolerance test (GTT). Busulfan depletion eliminated the majority of circulating blood monocytes, and recipients of uninfected and infected BM were equally reconstituted at 1-week post transfer (S3A Fig). By 8-weeks post reconstitution, recipients of BM from infected mice have a significantly higher frequency of circulating Ly6c^high^ monocytes as compared to recipients of uninfected BM (S3B Fig), mimicking the situation of intact infection, where infected mice have a higher frequency of blood monocytes than uninfected mice. Mice that received bone marrow from infected males have a significantly lower glucose area under the curve (AUC, Fig 5A and 5B) than those that received control bone marrow, indicating that systemic glucose metabolism can be modulated via hematopoietic cell transfer. We harvested bone marrow from all recipients and differentiated BMDM in M-CSF for 6 days and then performed real-time extracellular flux analysis. (Fig 5C). BMDM from recipients of bone marrow from *S*. *mansoni* infected mice had significantly higher basal oxygen consumption and a trend towards increased spare respiratory capacity as compared to BMDM generated from recipients of uninfected control bone marrow (Fig 5D and 5E). Additionally, BMDM from recipients of bone marrow from *S*. *mansoni* infected mice had a significantly higher Mitotracker MFI than BMDM from recipients of control bone marrow (Fig 5F), mimicking the increases seen in BMDM from patently infected males. These data strongly suggest that *S*. *mansoni* induced metabolic modulation of the myeloid lineage in males is long-lived even in the absence of ongoing exposure to egg antigens, and for the first time indicate that hematopoietic cells are at least partially responsible for the regulation of whole-body glucose metabolism by *S*. *mansoni* infection in the HFD ApoE^-/-^ model.

### *S*. *mansoni* infection protects male mice, but not female mice, from obesity and glucose intolerance independently of systemic alternative macrophage activation

**Fig 6:**
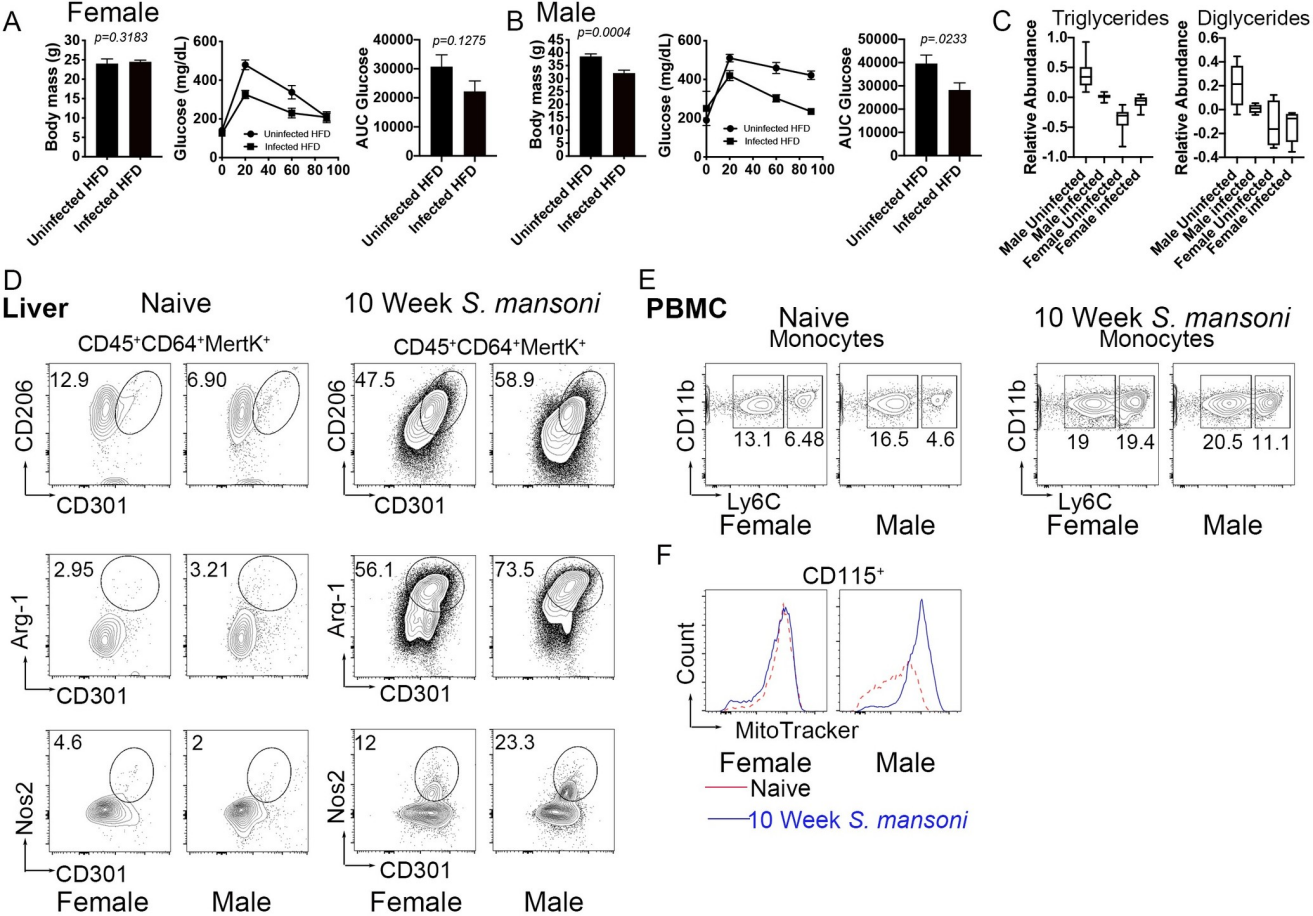
*S*. *mansoni* infection does not protect females from metabolic disease despite inducing alternative activation in hepatic macrophages. (A-B) Total body weight and glucose tolerance test (GTT) at 10 weeks post-infection. GTT values were analyzed by Area Under the Curve and graphed using GraphPad Prims. (C) Plots of relative abundance of serum di/triglycerides measured by LC-MS (D) Flow cytometry analysis of alternative activation markers CD206 and CD302 in perfused and digested livers gated on hepatic macrophages (CD45^+^CD64^+^MertK^+^) Arg-1 expression in hepatic macrophages from uninfected and infected ApoE^-/-^ mice Nos-2 expression by flow cytometry in hepatic macrophages 10 weeks post infection. (E) Ly6C expression in monocyte from peripheral blood mononuclear cells (PBMC) at 10-week post infection by flow cytometry (F) MitoTracker Red in CD115^+^ monocytes from PBMC at 10 weeks post immunization in uninfected and infected ApoE^-/-^ mice. Graphs are representative from experiments that were performed 3–4 times with n>4. Exception is MitoTracker data, which was performed twice, with n>4. Statistical analysis was done using unpaired Student’s t test, *p < 0.05; **p < 0.01.

Biological sex is a major contributor to cardiovascular and metabolic phenotypes in mammals [44], so we assessed the sex-dependent impact of *S*. *mansoni* infection on obesity and glucose intolerance. For this, we fed male and female ApoE^-/-^ HFD for 10 days before infection. We infected and mock infected mice (controls). Ten weeks post infection we analyzed body weight and glucose tolerance (via an IP glucose tolerance test) and found that infection is significantly beneficial for male, but not female mice, as only males are protected from HFD-induced weight gain and glucose intolerance (Fig 6A and 6B). We also analyzed serum triglyceride (TG) and diglyceride (DG) levels using untargeted lipidomics and found that relative abundance of both TG and DG were decreased in infected males as compared to uninfected males, while TG were increased by infection in females and DG were unchanged (Fig 6C). These data indicate that *S*. *mansoni* infection induces a sex-dependent modulation of systemic metabolic disease parameters. We then wondered if this infection-mediated effect in males only was correlated with differences in systemic alternative activation of macrophages in females. Flow cytometry analysis showed that alternative activation markers (CD206, CD301, Arg1) were highly expressed in hepatic (the liver is the major tissue site of egg deposition) macrophages from male and female mice following infection, but not in naïve mice (Fig 6D), suggesting that *S*. *mansoni* induced alternative activation irrespective of sex. Previous studies characterizing the dynamics of alternatively activated macrophages during schistosome infection have found that these macrophages largely arise from Ly6C^high^ monocytes [21, 45]. Naïve male and female ApoE^-/-^ mice on HFD had equivalent frequencies of Ly6c^high^ monocytes circulating in peripheral blood. At 10-weeks post infection we found an increased percentage of both Ly6C^int^ and Ly6C^high^ cells (Fig 6E) in both male and female mice compared to the mock infected controls, with the frequency of Ly6C^high^ cells in females 1.74 times that of males, suggesting either increased monopoiesis in females, or increased tissue recruitment in males. Since we had found increased mitochondrial MFI in BMDM from infected male mice, we asked whether circulating blood monocytes are similarly modulated. We observed an infection induced increase in mitotracker fluorescent intensity in monocytes from male ApoE^-/-^ mice on HFD, but not from females (Fig 6F). These data suggest that there is sex-specific increased mitochondrial activity following infection in the monocyte cell population.

### *S*. *mansoni* infection modulates bone marrow myeloid progenitors in a sex-dependent manner

**Fig 7:**
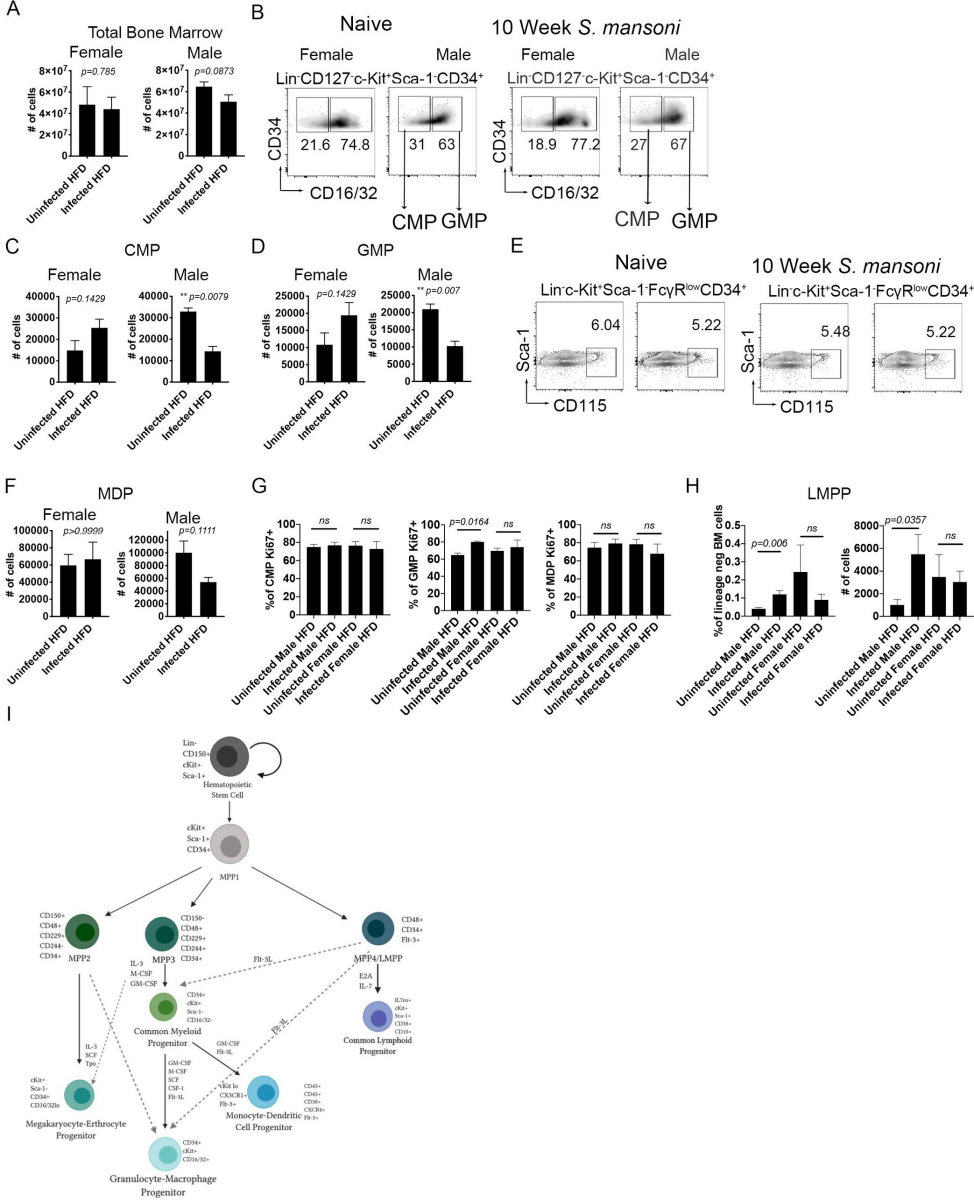
*S*. *mansoni* infection modulates bone marrow myeloid progenitors in a sex-dependent manner. (A) Total cell counts from bone marrow cells utilizing trypan-blue discrimination of apoptotic cells (B) Percentages of CMP (Lin^-^CD127^-^c-Kit^+^Sca-1^-^CD34^+^CD16/32^low^) and GMP (Lin^-^CD127^-^c-Kit^+^Sca-1^-^CD34^+^CD16/32^hi^) (C) CMP cell counts in bone marrow ApoE^-/-^ (D) GMP cell counts 10 week post infection in ApoE^-/-^ mice (E) Flow cytometry analysis of MDP defined as Lin^-^c-Kit^+^Sca-1^-^FcγR^low^CD34^+^CD115^+^ (F) MDP cell counts in ApoE^-/-^uninfected and infected male and female mice. (G) Ki67 expression in CMP, GMP, and MDP in bone marrow from 10-week infected and control male and female ApoE^-/-^ mice on HFD. (H) Frequency and total number of LMPP (defined as Lin^-^CD127^-^c-Kit^+^Sca-1^+^CD34^+^flt3^+^). (G) Diagram of mouse bone marrow progenitors/differentiation pathways. Graphs are representative from experiments that were performed 2–3 times with n>4. Statistical analysis was done using Brown Forsythe and Welch ANOVA.

Since we observed that *S*. *mansoni* infection modulation of blood monocytes is sex-dependent, we then wondered if these effects in the differentiated monocytes are the result of long-lasting changes in the myeloid lineage after helminth infection. For this, we analyzed the main lineages of hematopoietic progenitors that produce myeloid cells, particularly Ly6c^high^ monocytes [46]: granulocyte-monocyte progenitors (GMP), monocyte-DC progenitors (MDP) and the common myeloid progenitor (CMP) in female and male ApoE^-/-^ mice. CMP were defined as Lin^-^CD127^-^c-Kit^+^Sca-1^-^CD34^+^FcRII/III^lo/−^[47], GMP were defined as Lin^−^IL-7R^−^Sca-1^−^c-kit^+^CD34^+^FcR II/III^+^, and MDP were defined as defined as Lin^−^ c-Kit^+^ Sca-1^−^ CD34^+^ FcγR^lo^ CD115^hi^ cells. Overall bone marrow cellularity was not affected by schistosome infection (Fig 7A), however, the numbers of CMP and GMP in male infected mice were significantly reduced compared to uninfected controls, while GMP and CMP were not reduced in females (Fig 7B–7D). The numbers and percentages of MDP remained unchanged in infected females and males compared to uninfected controls (Fig 7E and 7F). Since overall numbers of CMPs and GMPs were modulated in males, we asked whether a higher percentage of these short-term progenitors were entering cell cycle. We found that equal frequencies of CMP and MDP in infected and uninfected control males and females were positive for Ki-67 (Fig 7G). In contrast, a significantly higher frequency of GMPs from infected males were Ki67^+^ as compared to uninfected controls, while female GMP were unchanged by *S*. *mansoni* infection (Fig 7G). Analyzing upstream multipotent progenitors, we found that *S*. *mansoni* infection increases the frequency and absolute number of LMMP (lymphoid primed multipotent progenitors) in males, but not females (Fig 7H). Traditionally GMPs have been thought to derive from CMPs, but recent reports have suggested that GMPs can differentiate directly from LMPP in a flt3 ligand dependent manner [48–50], with flt3 ligand overexpression biasing lymphoid-myeloid progenitors towards GMP differentiation [51]. A schematic of myeloid progenitor differentiation and the role of flt3 is depicted in Fig 7I. Our transcriptomic data in Fig 1 found a significant increase in flt3 transcripts in BMDM from infected males as compared to uninfected controls (0.675 LogFC, adjusted *p*-value = 3.10 x 10^−4^). These data strongly suggest that *S*. *mansoni* infection modulates both metabolic disease and myeloid differentiation from bone marrow progenitors in a sex-specific manner that is independent from the induction of systemic alternative activation.

## Discussion

Helminth infections in general, and schistosomiasis specifically, have been known to be inversely correlated with obesity and glucose intolerance for over a decade, a phenomenon thought to be associated with Type 2 polarization of macrophages and T cells. In the current study we report that *S*. *mansoni* infection induces dramatic metabolic alterations in BMDM from male ApoE^-/-^ mice on HFD. Our results indicate that macrophages derived from the bone marrow of infected male mice have increased basal oxygen consumption and spare respiratory capacity compared to those derived from uninfected males. In T cells, an increase in spare respiratory capacity has been linked to mitochondrial biogenesis, and controls the transition to a long-lived memory phenotype [52]. In macrophages, M2 (alternative) activation has previously been shown to lead to increased spare respiratory capacity, a process that also involves mitochondrial biogenesis [53], while TLR recognition of bacteria has been shown to increase mitochondrial respiration via modulation of complex I and II [54]. In both cell types, the increased mitochondrial respiration underlies the longevity of the cells. In our model we have not found most of the traditional markers used to define M2 alternative activation by flow cytometry at steady state (Fig 1). Arg1 is the only canonical M2 transcript that is modulated in the BMDM from infected male mice at steady state, and that fold increase is relatively low (.894 log FC, adjusted *p*-value = .035). Arg1 drives the production of polyamines, which in turn are able to modulate mitochondrial OxPHOS [55, 56]. In the currently accepted paradigm of M2 polarization STAT6 phosphorylation upregulates PGC1-β and PPARγ, leading to mitochondrial biogenesis and increased beta-oxidation in addition to Arg1transcription. Here we present a model where neither PGC1-β or PPARγ are transcriptionally modulated (mRNASeq data), but Arg1 transcription is increased with a concomitant dramatic increase in CPT-1 dependent mitochondrial respiration, suggesting that there may be alternative ways to modulate mitochondrial metabolism.

Alternative activation has previously been shown to be dependent on cell intrinsic lysosomal lipolysis and lysosomal acid lipase (lal), with the defining feature being reductions in lipid droplets [23]. In our model using unstimulated BMDM from *S*. *mansoni* infected mice there is no gross difference in lipid droplets, nor an increase in lal transcripts. Instead, we found a significant shift in the lipidome of BMDM from infected male mice that centered on a reduction in cholesterol esters, and a dramatic increase in free fatty acids. Previously published work has found that lipolysis centered on TGs as a fatty acid source in IL-4 induced M2 macrophages [23]. While we did find reductions in two species of DGs and one species TG, there were more significant reductions in CE and increases in free fatty acids, that suggest metabolism is centered on a combination of lipolysis and uptake of exogenous free fatty acids. The data that inhibition of mgll reduces both basal and max OCR supports the notion of increased lipolysis in this model, while our finding of increased ffar2 (a transporter of short chain fatty acids) and our heavy palmitate tracing and fatty acid oxidation seahorse assays where exogenous carbons are limited to palmitate, bolsters the idea that egg antigen exposure drives increased importation of exogenous fatty acids into macrophages. Since increases in plasma free fatty acids are associated with hyperglycemia and the induction of insulin resistance [57, 58], infection induced increases in macrophage uptake/usage of exogenous fatty acids may contribute to the observed improvements in glucose tolerance in this model, with tissue macrophages acting as a fatty acid sink. The lipidome of BMDM from IL-4c injected mice has reductions in some of the same CE seen in BMDM from infected males, but fewer species (8 as opposed to 12), and no reductions in TG, DG, nor an increase in free fatty acids, suggesting that CE reduction is not a sufficient biomarker of increased FAO in this model, and that the true correlate of modulation of systemic disease may be macrophage free fatty acids. Flux analysis with heavy carbon labeled glucose and palmitate indicate that BMDM from male infected mice have increased shuttling of both glucose and palmitate into TCA cycle intermediates, supporting the conclusion that these cells have increased mitochondrial beta oxidation, and raising the possibility that these BMDM may upregulate glycolysis to support the need for β-oxidation substrates, a possibility that will be explored in future work. In addition to the unique lipidomic modulations, we found that the dramatic increase in both basal oxygen consumption and spare respiratory capacity we observed in BMDM from male infected mice was significantly dependent on palmitate and CPT1 activity, as etomoxir dramatically reduces spare respiratory capacity. This observation further supports the idea that *in vivo* exposure of myeloid precursors to helminth antigens induces unique metabolic modulations that focus on cholesterol and lipid metabolism as a source for beta oxidation. These data suggest that hybrid metabolic states in the absence of overt M1 or M2 polarization occur in macrophages in the context of chronic helminth disease, and present a challenge to the dichotomy of M1 versus M2 polarization states being inextricably linked to immunometabolism. Instead, FAO may be necessary, but not sufficient to drive true M2 polarization. Future studies exploring the immunometabolism of bone marrow derived and tissue resident macrophages and dendritic cells from other chronic infections and inflammatory diseases are needed in order to obtain a true picture of the correlation between metabolism and myeloid polarization/function *in vivo*.

The data presented here are from BMDM that we believe represent the monocyte derived macrophage pool that is available for recruitment to aortic plaques, adipose tissue, and the liver. Macrophage cholesterol efflux to HDL is important for retarding/reversing foam cell formation in the plaque [59], so the reductions in multiple species of cholesterol esters is important. This reduction in CE species may be part of the mechanism through which *S*. *mansoni* improves aortic plaques [15], and will be the subject of future studies.

There are significant clinical differences in both the etiology and pathology of diabetes and cardiovascular disease between males and females [60, 61], but sex differences in immunological activation or modulation by *S*. *mansoni* infection have not previously been well studied in humans or animal models. Surprisingly, we found that *S*. *mansoni* infection does not reliably protect female ApoE^-/-^ mice from high fat diet induced obesity or glucose intolerance. Interestingly, hepatic macrophage alternative activation in response to infection is equivalent between males and females, but infection increases blood Ly6c^high^monocyte frequency in the females to a much greater extent than the males. Blood monocytes from infected males phenocopy the increase in Mitotracker MFI that we have found in BMDM generated from infected males, indicating that our BMDM model is likely an accurate representation of the *in vivo* potential of the myeloid compartment. Interestingly, we found that infection significantly reduced the total number of CMP and GMP in male, but not female bone marrow, again pointing to a sex-specific modulation of the myeloid lineage at the precursor level. The concomitant sex-specific increase in GMP Ki-67 and LMPP and decrease in total number of GMPs suggests either sex-dependent infection driven modulation of GMP differentiation into monocytes, or reduced lifespan of GMPs. Future work will focus on carefully delineating these mechanisms.

Our data indicate that *S*. *mansoni* infection induces a hybrid inflammatory state in male BMDM, where the LPS induced production of nitrite and iNos is enhanced, while the production of the key chronic pro-inflammatory mediators IL-12p70, CXCL1, and IL-6 are reduced. This inflammatory profile is distinct from what has previously been published with IL-4 induced M2 macrophages, where the production of iNos and nitrite are reduced following TLR stimulation [62], and is unique from what we observe in mice chronically administered IL-4 complexes, where neither iNos nor nitrite are significantly altered as compared to control BMDM (Fig 4). CXCL1 and IL-6 have previously been linked to increased monocyte recruitment and disease progression in both atherosclerosis and obesity-induced diabetes [63–67], so these data support the possibility of infection driven modulation of macrophage function supporting the decreased pathology seen in infected males. We have demonstrated that the modulation of macrophage oxygen consumption is transferrable to an uninfected recipient, and can last for at least ten weeks, suggesting that, *S*. *mansoni* infection induces long-lived metabolic modulation of the myeloid lineage that survives in the absence of ongoing antigenic exposure. Trained innate immunity has previously been documented to be induced by BCG immunization [68], and has recently been suggested to be the mechanism underlying the association between previous bacterial and fungal infections and the development of atherosclerosis [69, 70]. In these models, trained immunity and epigenetic reprogramming is driven in part from a switch from oxidative phosphorylation to increased aerobic glycolysis [71, 72]. In the case of BCG, trained circulating monocytes can be found months after immunization, which strongly suggests reprogramming of bone marrow progenitors [73]. Recent reports indicate that western high-fat diet itself also induces innate training of bone marrow progenitors in both the Ldr^-/-^ model of atherosclerosis [74] and in obesity related steatohepatitis [75]. Our data suggests that *S*. *mansoni* infection in male mice trains the myeloid lineage in the opposite fashion; modulating the metabolic transcriptome of the myeloid lineage such that oxidative phosphorylation and mitochondrial activity is increased, while the chronic inflammatory potential is decreased. The down-regulation of Mthfr and the one carbon folate pathway, in addition to our data indicating increased carbon shuttling into succinate suggest that *S*. *mansoni* trained immunity may also be regulated via progenitor epigenetic reprogramming, a possibility bolstered by the transferability of both increased glucose tolerance and the increased BMDM mitochondrial respiration via bone marrow from infected males. Our finding of increased flt3 transcript in BMDM, along with increased LMPPs in the bone marrow of infected males support modulation at least at the LMPP level. Future work will focus on the genetic mechanism/s of schistosome induced myeloid reprogramming and the role that biological sex plays in reprogramming.

## Materials and methods

### Ethics statement

This study was carried out in accordance with the recommendations in the Guide for the Care and Use of Laboratory Animals of the National Institutes of Health. The protocols were approved by the Institutional Animal Care and Use Committees of the University of Utah (#18–09001) and Purdue University (1406001081A001).

### Parasite and mouse models

Snails infected with *S*. *mansoni* (strain NMRI, NR-21962) were provided by the Schistosome Research Reagent Resource Center for distribution by BEI Resources, NIAID NIH. ApoE^-/-^ (B6.129P2-Apoetm1Unc/J) were purchased from the Jackson Laboratories and bred at the University of Utah. 6-8-week-old male mice were housed in pathogen-free conditions and were fed standard rodent chow (2019 rodent chow, Harlan Teklad) until 10–14 days before infection when they were transitioned to a high-fat diet (HFD: 21% milk fat, 0.15% cholesterol: TD 88137 Envigo). Bone marrow chimeras were generated by treating male ApoE^-/-^ mice that had been on high- fat diet for 4 weeks with 20mg/kg of pharmaceutical grade busulfan for 5 days (total dose of 100mg/kg). On day 6 mice were i.v. injected with 2.5–3 x 10^6^ bone marrow cells from either 10-week *S*. *mansoni* infected or control uninfected ApoE^-/-^ mice on high-fat diet. Reconstitution was validated via flow-cytometry at 3-weeks post-transfer and recipient mice were maintained on high-fat diet for 10-weeks post-reconstitution.

### *S*. *mansoni* infection and glucose tolerance test

ApoE^-/-^ male mice of 6 weeks of age were exposed percutaneously to 75–90 cercariae of *S*. *mansoni* or were mocked infected (as controls). At five- and ten-weeks post-infection mice were fasted for five hours and baseline blood glucose levels were obtained via lateral tail vein nick. Mice were then administered a single intraperitoneal injection of glucose (2mg/g of body weight, ultrapure glucose, Sigma G7528). Blood glucose levels were obtained at 20, 60, and 90-minutes post injection. Individual data points obtained were analyzed by Area Under Curve (AUC).

### IL-4 complex treatment

ApoE^-/-^ male mice were fed HFD and 4–5 weeks later they were injected I.P. with complexed IL-4, composed of IL-4 (Shenandoah Biotechnology, 5 μg) mixed with IL-4 (BioXcell, 25 μg) dissolved in phosphate buffered saline (PBS) every 2–3 days for 4.5–5 weeks. Control mice were injected with an equivalent volume of PBS.

### Mouse macrophage culture

Mouse bone marrow-derived macrophages (BMDM) were generated as follows: bone marrow cells were isolated by centrifugation of bones at >10,000 x g in a microcentrifuge tube for 15 seconds as previously described [76]. Cells were differentiated in M-CSF (20ng/mL, Peprotech, Rocky Hill, NJ) in complete macrophage medium (CMM: RPMI1640, 10% FCS, 2mM L-glutamine and 1 IU/mL Pen-Strep for 6 or 7 days. On the last day, cells were harvested in Cellstripper cell dissociation reagent (Corning) were washed with CMM and prepared for downstream assays.

### Glycolytic and phospho-oxidative metabolism measurement (seahorse assay)

BMDM from different conditions (uninfected controls or *S*. *mansoni* infected) were resuspended at the same concentration in XF assay media supplemented with 5% FCS and 5mM glucose. The day before the assay, the probe plate was calibrated and incubated at 37°C in a non-CO_2_ incubator. Resuspended cells were seeded at a concentration of 1.5x10^5^ cells per well and incubated for 20–60 minutes in the Prep Station incubator (37°C non-CO_2_ incubator). Following initial incubation, XF Running Media (XF assay media with 5% FCS and 10mM Glucose) were dispensed into each well. OCR and ECAR were measured by an XF96 Seahorse Extracellular Flux Analyzer following the manufacturer’s instructions. For the seahorse assay, cells were treated with oligomycin (1μM), FCCP (1.5μM), rotenone (100nM) and antimycin A (1μM). Each condition was performed in 2–3 technical replicates. For determination of palmitate dependent respiration, BSA-conjugated palmitate (BSA: palmitate = 1:6, molar ratio) was prepared according to the Seahorse protocol (Seahorse Bioscience). Briefly, 1 mM sodium palmitate (Sigma Aldrich) was conjugated with 0.17 mM fatty acid free-BSA (Sigma Aldrich) in 150 mM NaCl solution at 37°C for 1h. Palmitate-BSA was stored in glass vials at -20°C until use. Cells were incubated as above in glucose limited XF media per manufacturer instructions.

### Mgll-dependent Mitochondrial Respiration Measurements

To assess Mgll-dependent mitochondrial respiration, BMDM were differentiated as above and harvested at Day 7. Cells were plated at 2 x 10^5^ cells/well into 96-well Seahorse cell culture dishes in RPMI with 10% FCS, 5 mM glucose, and 2 mM L-glutamine in the presence or absence of 1 μM Mgll inhibitor JZL 184 (Tocris). Prior to extracellular flux analysis, media was changed to running media (XF RPMI, 5% FCS, 5 mM glucose, 2 mM L-glutamine, and DMSO or inhibitor as indicated). A standard mitochondrial stress test was performed as above. Basal Respiration was measured as the average resting Oxygen Consumption Rate (OCR) of cells prior to oligomycin injection. Maximal Respiration was measured as the average OCR of cells following treatment with mitochondrial gradient uncoupler FCCP.

### Flow cytometry

Bone marrow cells were obtained by centrifugation of bones into tubes at >10000 rpm for 15 s and cultured for 6–7 days in M-CSF (20ng/mL, Peprotech, Rocky Hill, NJ) in complete macrophage medium (CMM: RPMI1640, 10% FCS, 2mM L-glutamine and 1 IU/mL Pen-Strep for 6 or 7 days. Surface staining of unstimulated BMDM was performed using the following mAb against mouse antigens: CD45 (Clone 30-F11,Biolegend), CD301(BioRad), CD206 (Clone: C06C2, Biolegend), F4/80 (BM8, Biolegend), CD64 (X54-5/7.1, BD), mouse Mer (Mertk) biotinylated (R&D). Intracellular antigen staining such as Nos2 (Clone: CXNFT, Invitrogen) and Arg1 (Clone: A1exF5, Invitrogen) was performed using the Intracellular Fixation and Permeabilization Buffer set (Thermo Fisher Scientific cat. no. 88–8824) per manufacturer’s instruction. Further, bone marrow cells were obtained by centrifugation of bones into tubes at >10000 rpm for 15 s. Surface staining for bone marrow precursors was performed using the following antibodies: Ter119 (Clone: Ter-119, Invitrogen), CD19 (Clone: MB19-1 Invitrogen), CD4 (Clone: GK1.5, Biolegend), CD3 (Clone: 17A2, Biolegend), Gr-1 (Clone: RB6-8C5, BD), CD11b (Clone: M1/70), Sca-1 (Clone: D7, eBiosciences), CD115 (Clone: AFS98, eBiosciences), Ly6C (Clone: HK1.4, Invitrogen), CD117/c-Kit (R&D), CD135/flt3 (Clone: A2F10.1, BD), CD127 (Clone: A7R34, Biolegend), CD34 (Clone: SA376A4 Biolegend) and CD16/32 (Clone: 93, Invitrogen). PBMC from whole blood were obtained following red blood cell lysis and used for flow cytometry analysis. Surface staining of PBMC was performed using Ter119, CD64, CD11b, CD115, Ly6C and MitoTracker Red.

Samples were acquired using Attune NxT Focusing Flow Cytometer (Thermo Fisher Scientific) and analyzed using Flowjo X 10.0.7r2 (FlowJo LLC, Inc.).

### Measurement of Cytokines and Inflammatory mediators

For cytokine levels of BMDCs, supernatants were collected at 24 hours post stimulation and measured with Mouse Cytokine and Chemokine ProcartaPlex 26plex panel (Life Technologies) per manufacture instructions using a Luminex Magpix system. Nitrite levels in cell culture media were determined using a Griess reagent kit for nitrite determination (Invitrogen) according the manufacturer's instructions.

### Heavy glucose and heavy palmitate labeling

For metabolomics tracing in Fig 2 BMDM were differentiated in CMM containing normal glucose. At Day 6 of culture cells were switched to CMM containing ^13^C_6_-glucose (Santa Cruz Biotech) for 24 hours. Cells were harvested and processed as described below. For metabolomics tracing in Fig 3 BMDM were differentiated in CMM containing normal glucose and serum. At Day 6 of culture cells were switched to CMM containing dialyzed serum and 1 mM ^13^C palmitate (Cambridge Isotope Laboratories) for 36 hours. Cells were harvested and processed as described above.

### RNA Sequencing

#### Library Preparation and sequencing

The concentration and quality of total RNA samples was first assessed using Agilent 2100 Bioanalyzer. A RIN (RNA Integrity Number) of five or higher was required to pass the quality control. Then 200ng of RNA per sample were used to prepared dual-indexed strand-specific cDNA library using KAPA mRNA Hyperprep Kit (Roche). The resulting libraries were assessed for its quantity and size distribution using Qubit and Agilent 2100 Bioanalyzer. Two hundred pico molar pooled libraries were utilized per flowcell for clustering amplification on cBot using HiSeq 3000/4000 PE Cluster Kit and sequenced with 2.75bp paired-end configuration on HiSeq4000 (Illumina) using HiSeq 3000/4000 PE SBS Kit. A Phred quality score (Q score) was used to measure the quality of sequencing. More than 90% of the sequencing reads reached Q30 (99.9% base call accuracy).

### RNASeq sequence alignment, gene counts, pathway analysis

The sequencing data were first assessed using FastQC (Babraham Bioinformatics) for quality control. Then all sequenced libraries were mapped to the mouse genome (UCSC mm10) using STAR RNA-seq aligner [77] with the following parameter: “—outSAMmapqUnique 60”. The reads distribution across the genome was assessed using bamutils (from ngsutils) [78]. Uniquely mapped sequencing reads were assigned to mm10 refGene genes using featureCounts (from subread) [79] with the following parameters: “-s 2 –p–Q 10”. Quality control of sequencing and mapping results was summarized using MultiQC [80]. Genes with read count per million > 0.5 in more than 2 of the samples were kept. The data was normalized using trimmed mean of M values method. Differential expression analysis was performed using edgeR [81, 82]. False discovery rate was computed from p-values using the Benjamini-Hochberg procedure.

The data (biological pathways, processes and interactions.) were analyzed using Advaita Bio's iPathwayGuide (http://www.advaitabio.com/ipathwayguide). Pathway analysis was performed on log_2_-transformed data using Bonferroni-corrected *p*-values. The data discussed in this publication have been deposited in NCBI's Gene Expression Omnibus [83] and are accessible through GEO Series accession number GSE155175 (https://www.ncbi.nlm.nih.gov/geo/query/acc.cgi?acc=GSE155175)

### RNA Isolation and q-RT-PCR

BMDM were stored in Trizol, and RNA isolation was performed as described in the Immunological Genome Project Total RNA isolation protocol. Next, cDNA was synthesized from RNA using Superscript IV VILO (ThermoFisher Scientific) for reverse transcription. qPCR was performed using TaqMan Gene expression assays (mgll, slc1a3, beta actin, ThermoFisher) on an Applied Biosystems Stepone Plus Real-Time PCR System. Beta-Actin assay number Mm00607939_s1, mgll assay Mm00449274_m1, slc1a3 assay Mm00600697_m1. Relative expression was calculated using the 2-ΔΔCt method [84].

### Untargeted lipidomics

#### Sample extraction from serum or cell pellets

Lipids were extracted from serum (50μL) or cell pellets in a combined solution as described by Matasy et al [85]. In detail, samples were incubated in solution with 225 μL MeOH containing internal standards (IS; Avanti SPLASH Lipidomix (Lot #12), 10μL per sample) and 750 μL methyl *tert*-butyl ether (MTBE). The samples were sonicated for 1 minutes, rested on ice for 1 hour, briefly vortexed every 15 minutes then an addition of 200μL dd-H2O was made to induce phase separation. All solutions were pre-chilled on ice. The sample were then vortexed for 20 s, rested at room temperature for 10 minutes, and centrifuged at 14,000 g for 10 minutes at 4 C. The upper organic phase was collected and evaporated to dryness under vacuum. Lipid samples were reconstituted in 200μL IPA and transferred to an LC-MS vial with insert for analysis. Concurrently, a process blank sample and pooled quality control (QC) sample was prepared by taking equal volumes (10μL per sample) from each sample after final resuspension.

#### LC-MS methods

Lipid extracts were separated on a Waters Acquity UPLC CSH C18 1.7 μm 100x2.1 mm column maintained at 65°C connected to an Agilent HiP 1290 Sampler, Agilent 1290 Infinity pump and Agilent 1290 Flex Cube and Agilent 6530 Accurate Mass Q-TOF dual ESI mass spectrometer. For positive mode, the source gas temperature was set to 225°C, with a gas flow of 11 L/minutes and a nebulizer pressure of 50 psig. VCap voltage was set at 3500 V, fragmentor at 110 V, skimmer at 85 V and Octopole RF peak at 750 V. For negative mode, the source gas temperature was set at 325°C, with a drying gas flow of 12 L/minutes and a nebulizer pressure of 30 psig. VCap voltage is set 3000 V, fragmentor at125 V, skimmer at 75 V and Octopole RF peak at 750 V. Reference masses in positive mode (*m/z* 121.0509 and 922.0098) were infused with nebulizer pressure at 2 psig, in negative mode reference masses (*m/z* 966.0007 and 112.9856) were infused with a nebulizer pressure at 5psig. Samples were analyzed in a randomized order in both positive and negative ionization modes in separate experiments acquiring with the scan range m/z 100–1700. Mobile phase A consisted of ACN:H_2_O (60:40 *v/v*) and mobile phase B consisted of IPA:ACN:H_2_0 (90:9:1 *v/v*), both contained 10 mM ammonium formate and 0.1% formic acid. The chromatography gradient for both positive and negative modes started at 15% mobile phase B then increased to 30% B over 2.4 minutes, then increased to 48% from 2.4–3.0 minutes, followed by an increase to 82% B from 3–13.2 minutes, and then to 99% from 13.2–13.8 minutes where it was held until 15.4 minutes and then returned to the initial conditioned and equilibrated for 4 min. Flow was 0.5 mL/minutes throughout, injection volume was 5μL for positive and 7 μL negative mode. Tandem mass spectrometry is conducted using the same LC gradient at collision energies of 20 V and 40 V. The pooled QC samples and process blank samples were injected throughout the sample queue to ensure the reliability of acquired LC-MS data.

### Lipid data analysis

Results from liquid chromatography-mass spectrometry (LC-MS) experiments were collected using Agilent MassHunter (MH) Workstation and analyzed using the software packages MH Qual, MH Quant (Agilent Technologies, Inc) and LipidMatch [86] to prepare the data set. The data table exported from MHQuant was evaluated using Excel where initial lipid targets were parsed based on the following criteria. Only lipids with relative standard deviation (RSD) less than 30% in the pooled quality control (QC) samples were used for data analysis. Additionally, targets identified in blanks or double blanks at significant amounts (area under the curve (AUC) target blank/AUC target QC >30%) were removed from analysis. Lipids were quantitated based on peak area ratios to the spiked internal standard (IS) of the same or nearest class. For relative abundance calculations, data were first normalized to the spiked IS concentration. The experimental data tables were then put into MetaboAnalyst and: 1) normalized to sum, 2) log transformed, and 3) treated with Pareto scaling if the target class contained more than one species.

### Metabolomics

#### Extraction

A cold 90% MeOH solution was added to each sample to give a final concentration of 80% MeOH to each cell pellet. Samples were incubated at -20°C for 1 hr. After incubation, the samples were centrifuged at 20,000 x g for 10 minutes at 4°C. The supernatant was transferred from each sample tube into a labeled, fresh micro centrifuge tube. The samples were dried *en vacuo*.

#### Mass spectrometry analysis of samples

All gas chromatography-mass spectrometry (GC-MS) analysis was performed with an Agilent 7200 GC-QTOF and an Agilent 7693A automatic liquid sampler. Dried samples were suspended in 40 μL of a 40 mg/mL O-methoxylamine hydrochloride (MOX) (MP Bio) in dry pyridine (EMD Millipore) and incubated for one hour at 37°C in a sand bath. 25 μL of this solution was added to auto sampler vials followed by the automatic addition of 60 μL of N-methyl-N-trimethylsilyltrifluoracetamide (MSTFA with 1%TMCS, Thermo) and incubated for 30 min at 37°C. Following incubation, each sample were vortexed and 1 μL of the prepared sample was injected into the GC inlet in the split mode with the inlet temperature held at 250°C. A 10:1 split ratio was used for analysis for the majority of metabolites. Any metabolites that saturated the instrument at the 10:1 split was analyzed at a 50:1 split ratio. The GC had an initial temperature of 60°C for one minute followed by a 10°C/min ramp to 325°C and a hold time of 10 min. A 30-meter Agilent Zorbax DB-5MS with 10 m Duraguard capillary column was employed for chromatographic separation. Helium was used as the carrier gas at a rate of 1 mL/min.

#### Data analysis

The area under the curve for each isotope was extracted using MHQuant software (Agilent). This data was exported as a.csv file and isotopically corrected using an in house modified version of DeuteRater [87].

### Statistical analysis

Statistical analyses of data were performed using one-way ANOVA, a non-parametric Mann-Whitney test, or unpaired Student's t-test depending on the data distribution. P ≤ 0.05 were considered statistically significant. Analyses and graphing were performed using Prism (GraphPad v8.0) and R-language for statistical computing.

## Supporting information

S1 Fig *S*. *mansoni* infection induced modulation of BMDM Fatty acid oxidation is independent of mouse genetic background. 10-week infected male IL4RFl/FlCre^neg^ and age-matched uninfected control IL4RFl/FlCre^neg^ mice were sacrificed. BMDM were differentiated with M-CSF in a 7-day culture with normal glucose and serum, and then switched to glucose limited media with Palmitate-BSA as the carbon source followed by Seahorse analysis of OCR and SRC. (A) SeaHorse assay results for OCR in basal conditions and in response to mitochondrial inhibitors. (B) Quantification (in picomoles/minute) of the palmitate basal oxygen consumption of BMDM. (C) Quantification of the palmitate spare respiratory capacity of BMDM (D) Oil Red O relative staining in BMDM. (E) MitoTracker Red Deep Stain measured by flow cytometry in BMDM.
(TIF)

S2 Fig IL-4c administration induces in vivo alternative activation of macrophages, but induces a metabolic profile distinct from that of *S*. *mansoni* infection. ApoE^-/-^ mice were fed HFD diet for 10 days before infection with *S*. *mansoni*, or 5 weeks before injection with IL-4 complexes. A) peritoneal macrophages after 4.5 weeks of IL-4c injection. B-D) Bone marrow macrophages were differentiated with M-CSF and either total cellular lipids or total RNA isolated. B) PLS-DA derived score of LC-MS based lipidomic analysis of BMDM from HFD IL-4c injected and control males.(C, D). iPathway analysis showed distinct profiles in BMDM from C) *S*. *mansoni* infected, and D) IL-4 injected HFD ApoE^-/-^ mice.
(TIF)

S3 Fig Busulfan treatment depletes blood monocytes. ApoE^-/-^ mice were fed HFD diet for 4 weeks before myelodepletive busulfan treatment. A) Frequency of Ly6c^high^ monocytes in PBMC in intact control, un-reconstituted, and recipients of BM from uninfected and infected mice. B) Frequency of Ly6c^high^ monocytes in PBMC from recipients of uninfected control and infected BM
(TIF)
