# Supplementary figures and images for: Metabolic reprogramming of the myeloid lineage by Schistosoma mansoni infection persists independently of antigen exposure
Source: PLoS Pathog. 2021 Jan 8;17(1):e1009198. doi: 10.1371/journal.ppat.1009198 (PMC7819610; doi:10.1371/journal.ppat.1009198)

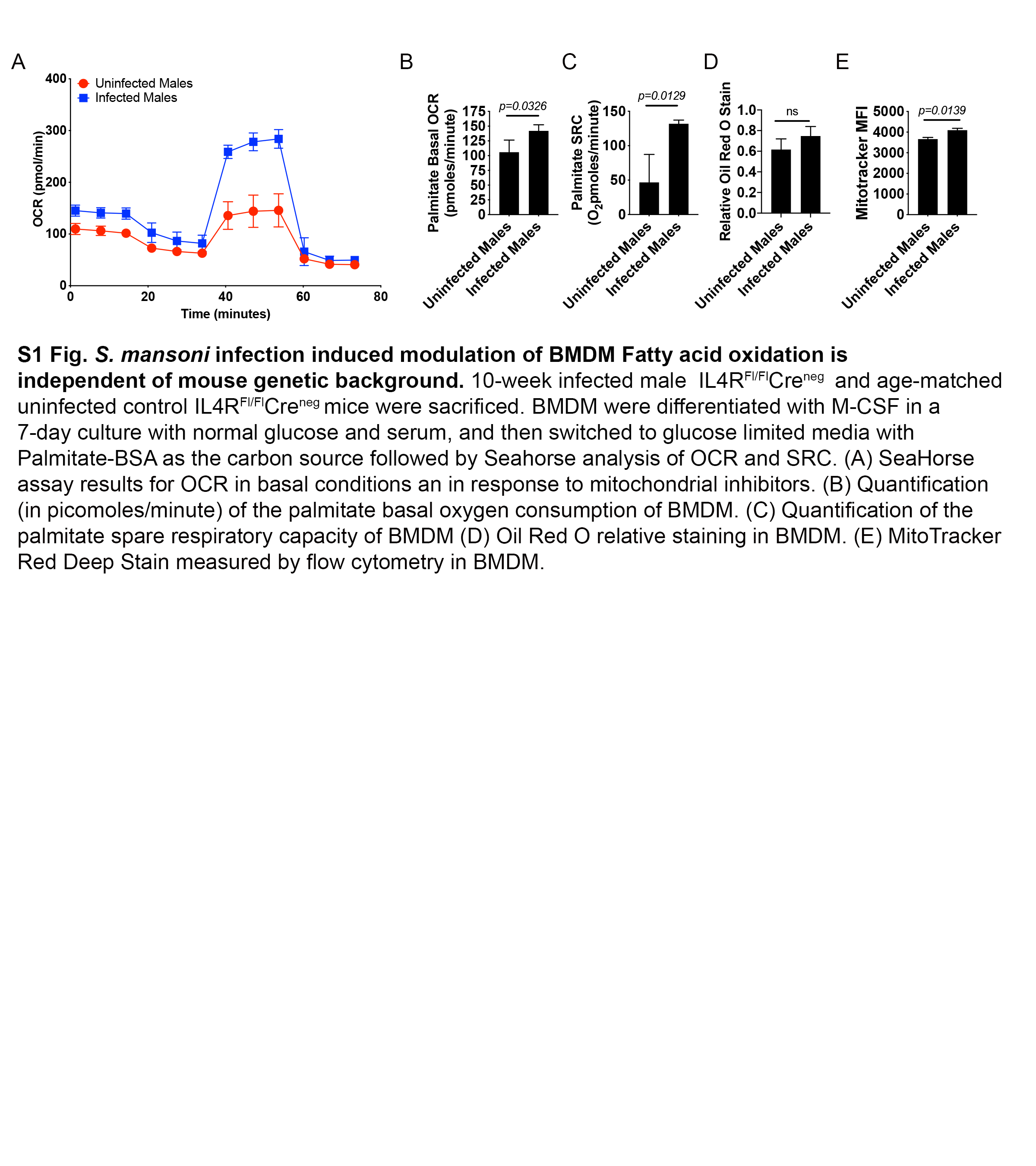

Supplement: S1 Fig — 10-week infected male IL4RFl/FlCreneg and age-matched uninfected control IL4RFl/FlCreneg mice were sacrificed. BMDM were differentiated with M-CSF in a 7-day culture with normal glucose and serum, and then switched to glucose limited media with Palmitate-BSA as the carbon source followed by Seahorse analysis of OCR and SRC. (A) SeaHorse assay results for OCR in basal conditions and in response to mitochondrial inhibitors. (B) Quantification (in picomoles/minute) of the palmitate basal oxygen consumption of BMDM. (C) Quantification of the palmitate spare respiratory capacity of BMDM (D) Oil Red O relative staining in BMDM. (E) MitoTracker Red Deep Stain measured by flow cytometry in BMDM. (TIF) [file ppat.1009198.s001.tif]

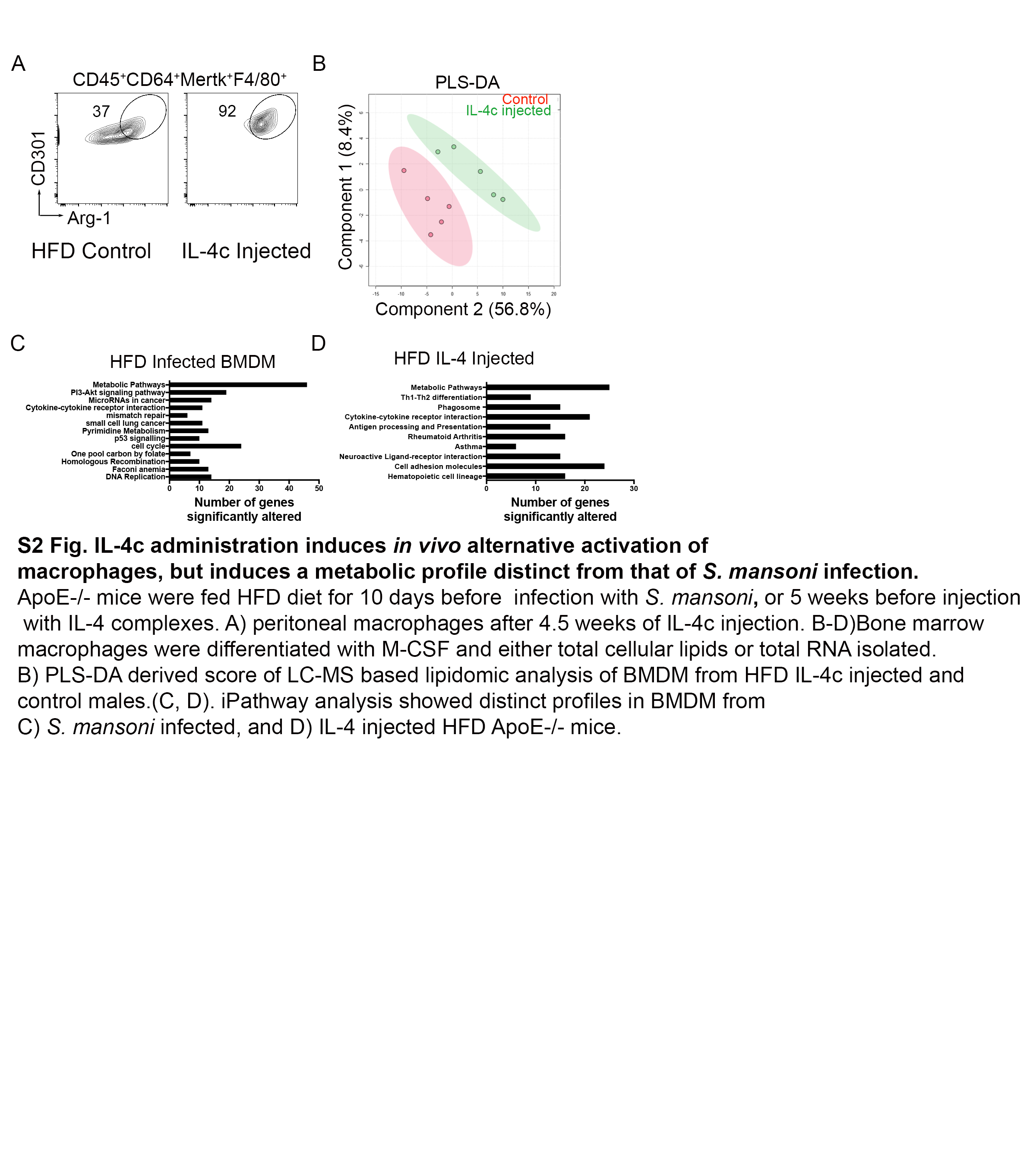

Supplement: S2 Fig — ApoE-/- mice were fed HFD diet for 10 days before infection with S. mansoni, or 5 weeks before injection with IL-4 complexes. A) peritoneal macrophages after 4.5 weeks of IL-4c injection. B-D) Bone marrow macrophages were differentiated with M-CSF and either total cellular lipids or total RNA isolated. B) PLS-DA derived score of LC-MS based lipidomic analysis of BMDM from HFD IL-4c injected and control males.(C, D). iPathway analysis showed distinct profiles in BMDM from C) S. mansoni infected, and D) IL-4 injected HFD ApoE-/- mice. (TIF) [file ppat.1009198.s002.tif]

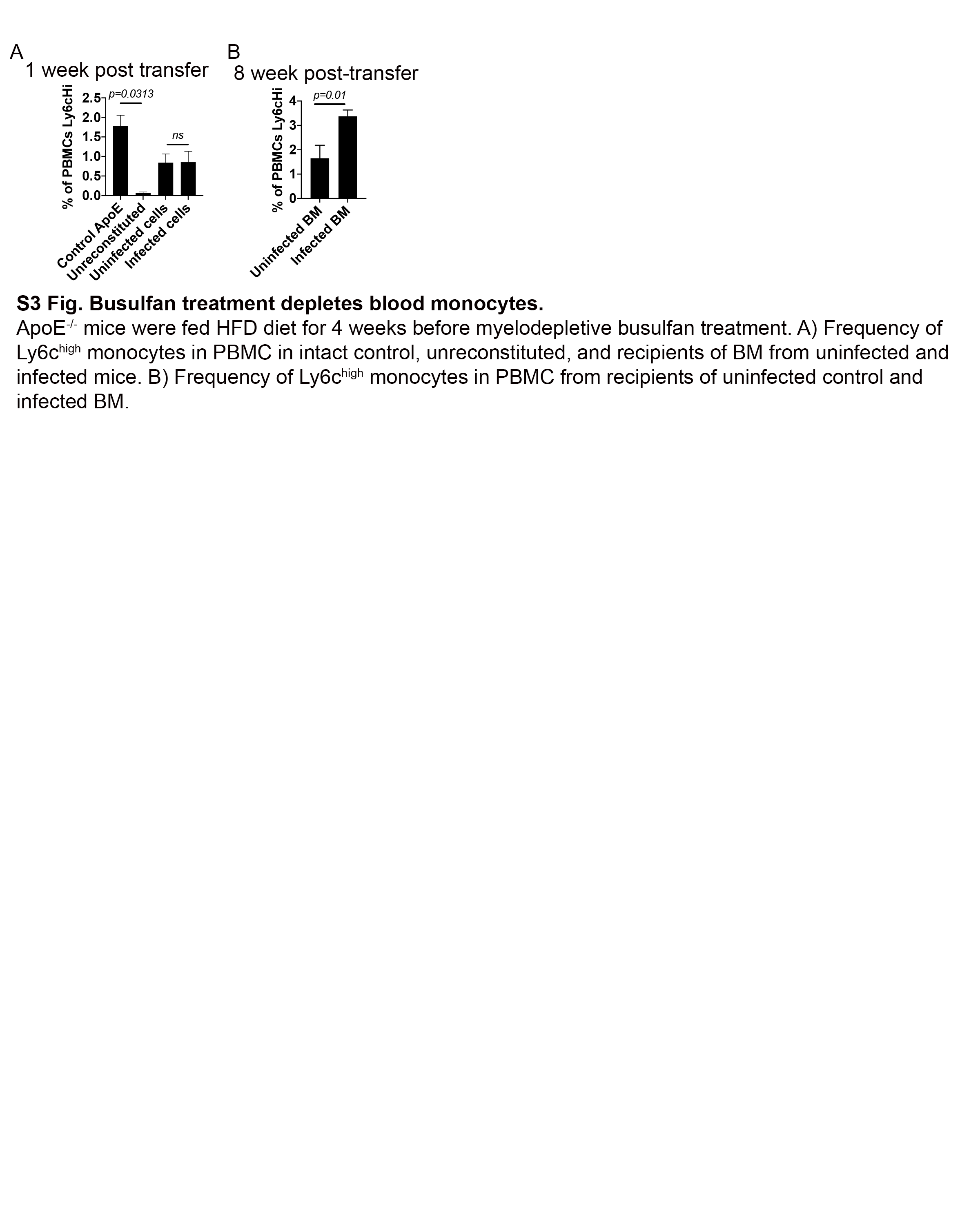

Supplement: S3 Fig — ApoE-/- mice were fed HFD diet for 4 weeks before myelodepletive busulfan treatment. A) Frequency of Ly6chigh monocytes in PBMC in intact control, un-reconstituted, and recipients of BM from uninfected and infected mice. B) Frequency of Ly6chigh monocytes in PBMC from recipients of uninfected control and infected BM (TIF) [file ppat.1009198.s003.tif]
